# Supplementary material for: Selective Sweeps and Polygenic Adaptation Drive Local Adaptation along Moisture and Temperature Gradients in Natural Populations of Coast Redwood and Giant Sequoia
Source: Genes (Basel). 2021 Nov 19;12(11):1826. doi: 10.3390/genes12111826 (PMC8621000; doi:10.3390/genes12111826)
Supplement: Supplementary file 1 [file genes-12-01826-s001.zip › Supplementary Figures.pdf]

GCAT TACG GCAT genes

MDPI

Supplementary Materials

Selective sweeps and polygenic adaptation drive local adaptation along moisture and temperature gradients in natural populations of coast redwood and giant sequoia

Amanda R De La Torre, Manoj K. Sekhwal, David B. Neale

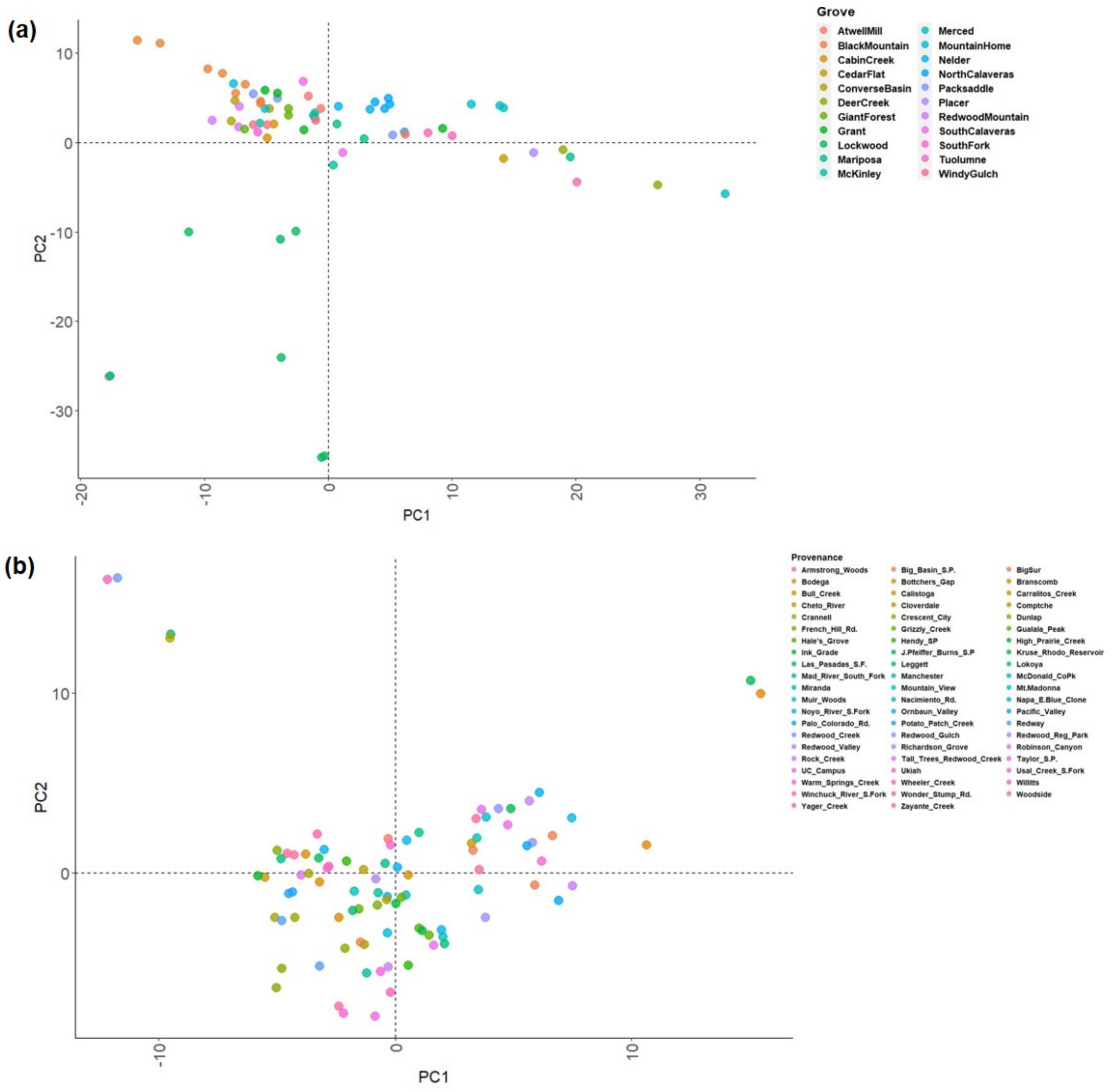

Supplementary Figure S1. Population structure in SEGI (a) and SESE (b) based on principal component analysis (PCA) results. PCA was carried out at TASSEL and plots were performed in R.

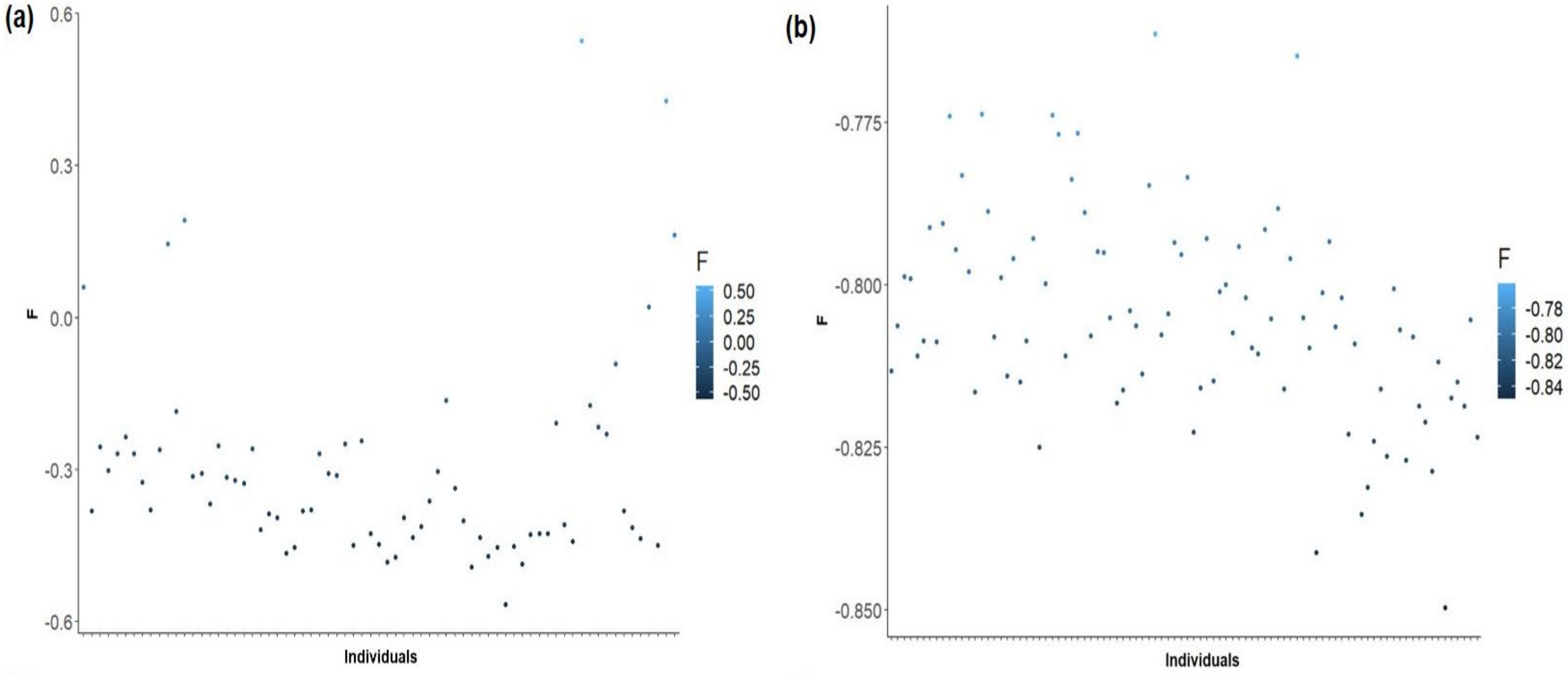
(a)
0.6
0.3
0.0
-0.3
-0.6
F
Individuals
F
0.50
0.25
0.00
-0.25
-0.50
(b)
-0.775
-0.800
-0.825
-0.850
F
Individuals
F
-0.78
-0.80
-0.82
-0.84

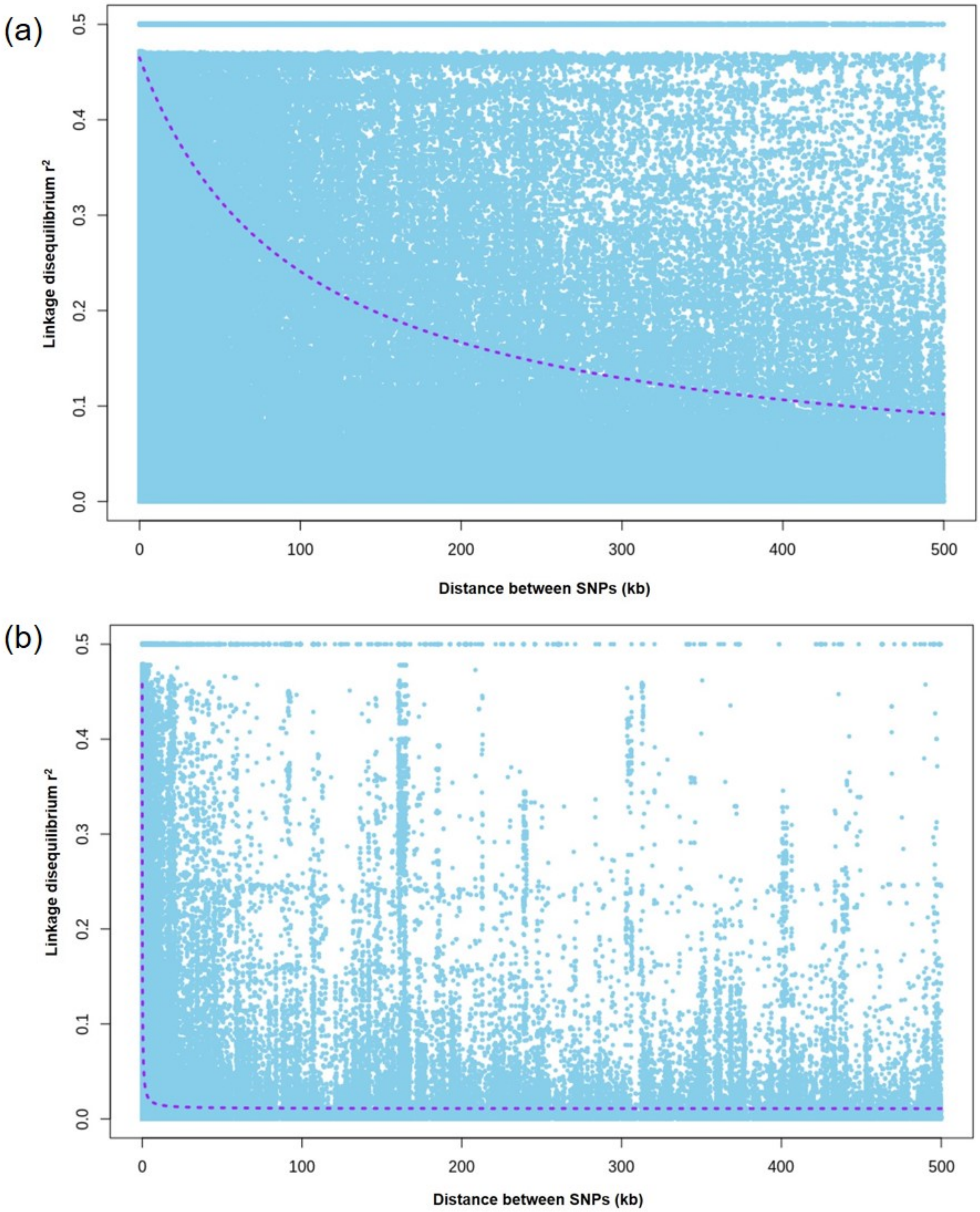
(a)
Linkage disequilibrium r²
Distance between SNPs (kb)
0.0
0.1
0.2
0.3
0.4
0.5
0
100
200
300
400
500
(b)
Linkage disequilibrium r²
Distance between SNPs (kb)
0.0
0.1
0.2
0.3
0.4
0.5
0
100
200
300
400
500

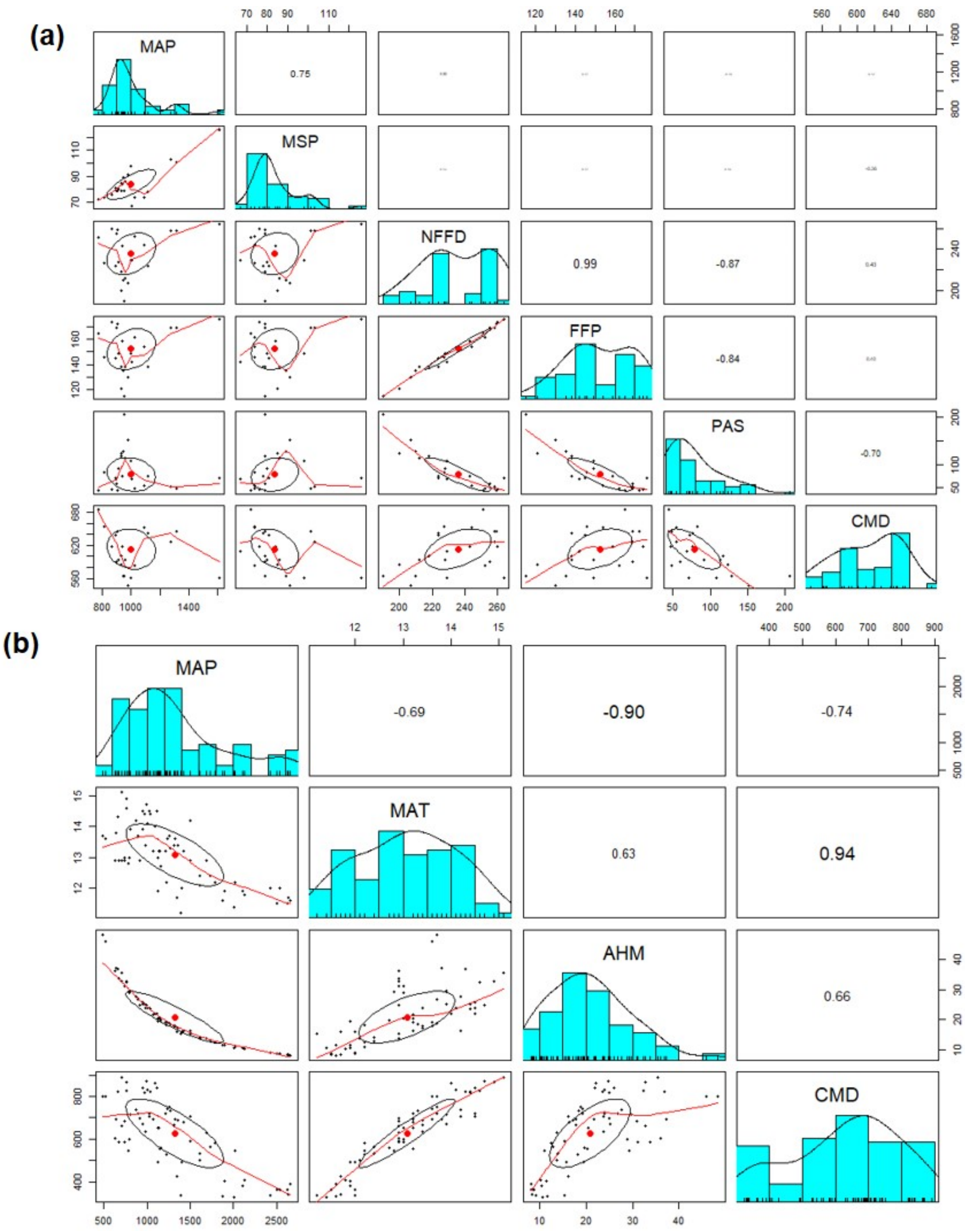
(a)
MAP
MSP
NFFD
FFP
PAS
CMD
0.75
0.99
-0.87
-0.84
-0.70
(b)
MAP
MAT
AHM
CMD
-0.69
-0.90
-0.74
0.63
0.94
0.66

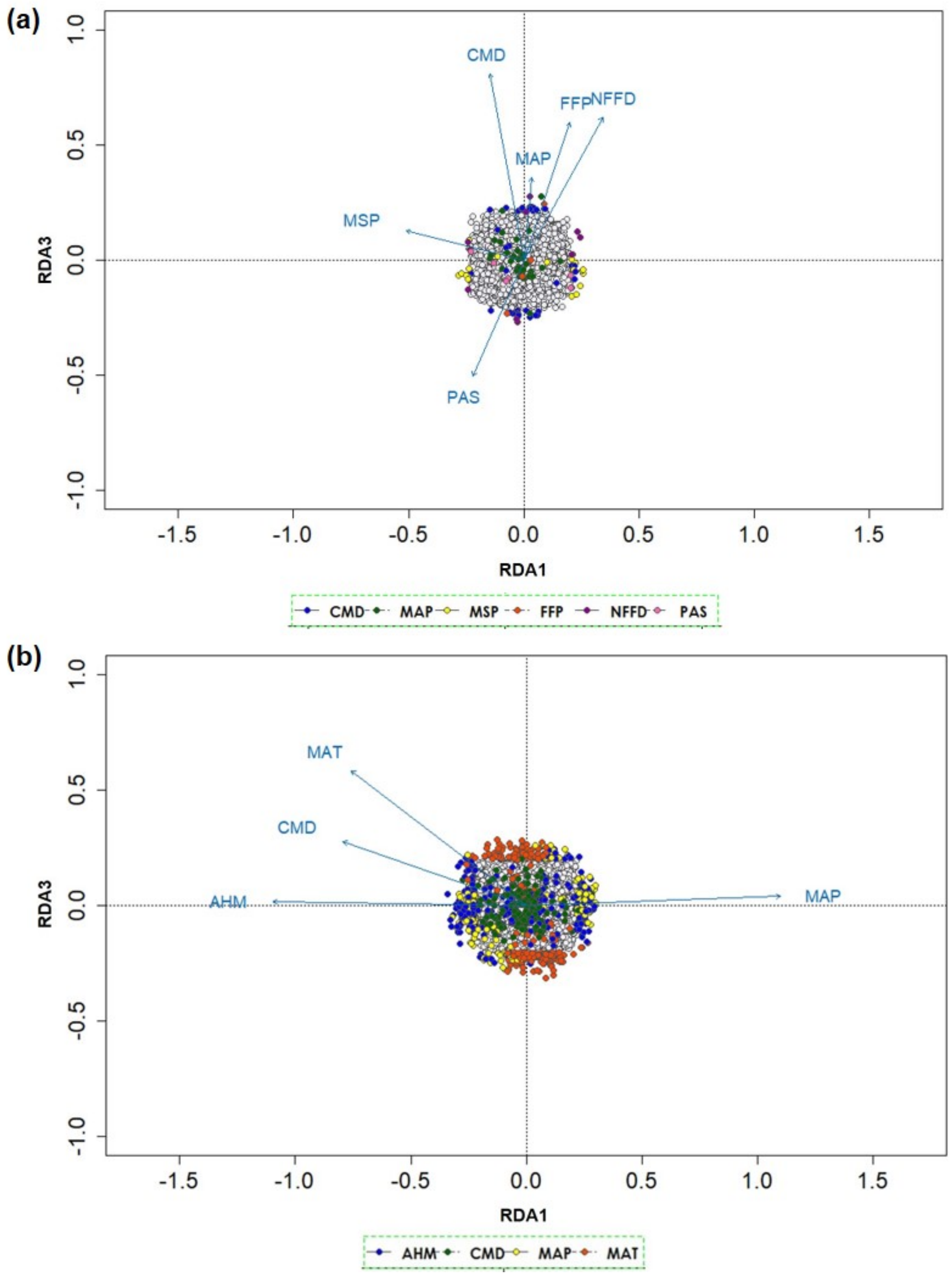
(a)
RDA3
RDA1
CMD
FFP
NFFD
MAP
MSP
PAS
1.0
0.5
0.0
-0.5
-1.0
-1.5
-1.0
-0.5
0.0
0.5
1.0
1.5
CMD MAP MSP FFP NFFD PAS
(b)
RDA3
RDA1
MAT
CMD
AHM
MAP
1.0
0.5
0.0
-0.5
-1.0
-1.5
-1.0
-0.5
0.0
0.5
1.0
1.5
AHM CMD MAP MAT

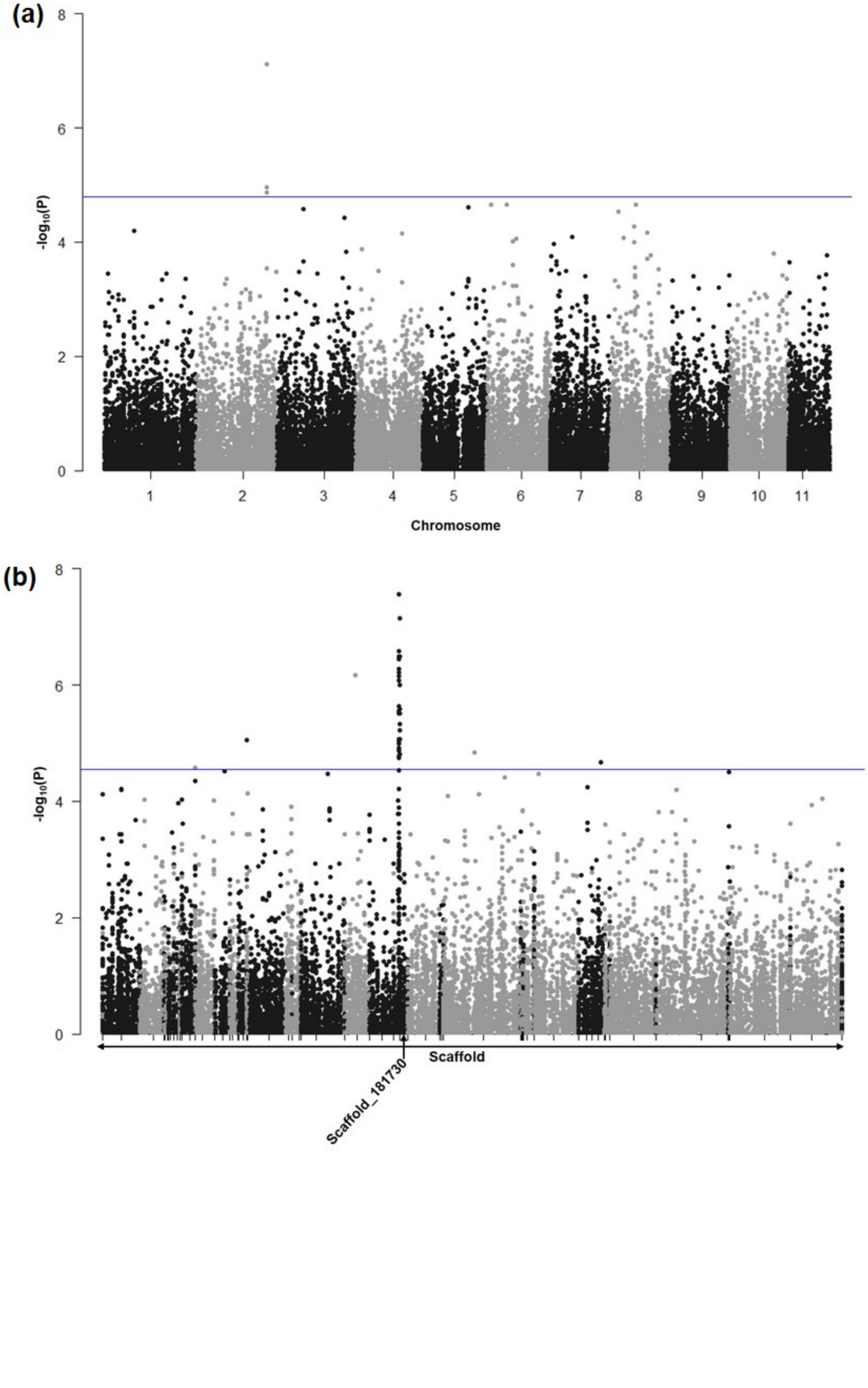
(a)
-log10(P)
8
6
4
2
0
1
2
3
4
5
6
7
8
9
10
11
Chromosome
(b)
-log10(P)
8
6
4
2
0
Scaffold
Scaffold_181730

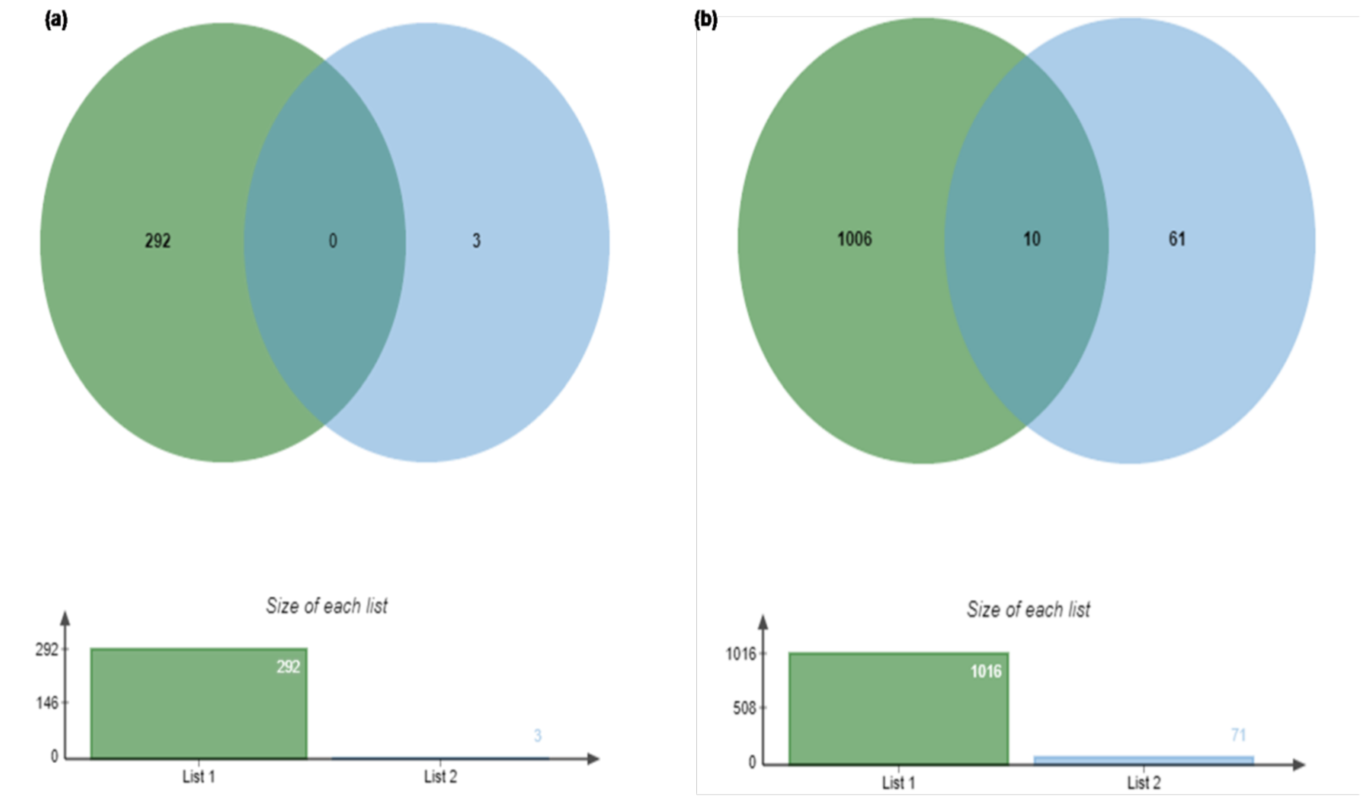
(a)
292
0
3
Size of each list
292
146
0
292
3
List 1
List 2
(b)
1006
10
61
Size of each list
1016
508
0
1016
71
List 1
List 2

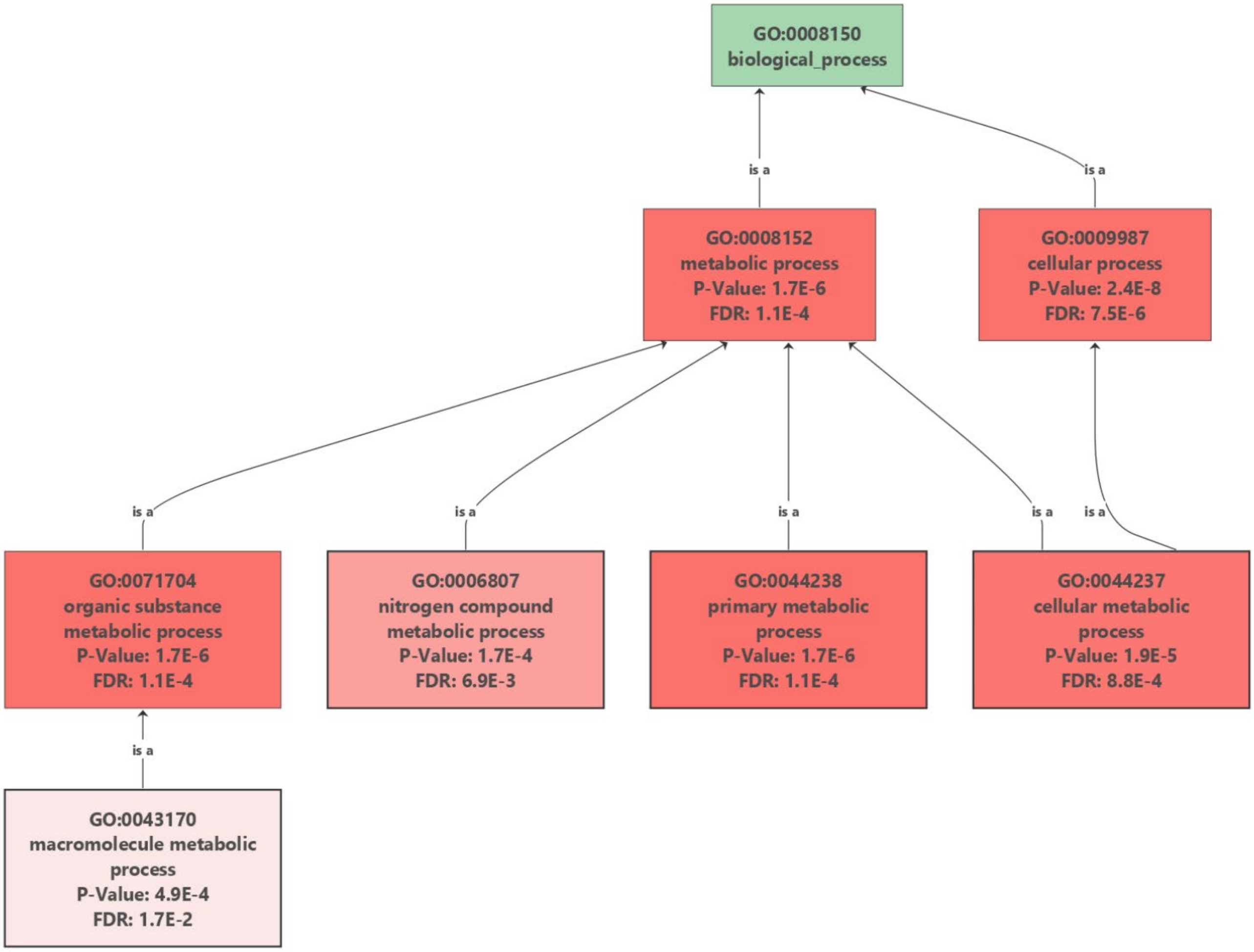

GO:0008150
biological_process
is a
is a
GO:0008152
metabolic process
P-Value: 1.7E-6
FDR: 1.1E-4
GO:0009987
cellular process
P-Value: 2.4E-8
FDR: 7.5E-6
is a
is a
is a
is a
is a
GO:0071704
organic substance metabolic process
P-Value: 1.7E-6
FDR: 1.1E-4
GO:0006807
nitrogen compound metabolic process
P-Value: 1.7E-4
FDR: 6.9E-3
GO:0044238
primary metabolic process
P-Value: 1.7E-6
FDR: 1.1E-4
GO:0044237
cellular metabolic process
P-Value: 1.9E-5
FDR: 8.8E-4
is a
GO:0043170
macromolecule metabolic process
P-Value: 4.9E-4
FDR: 1.7E-2

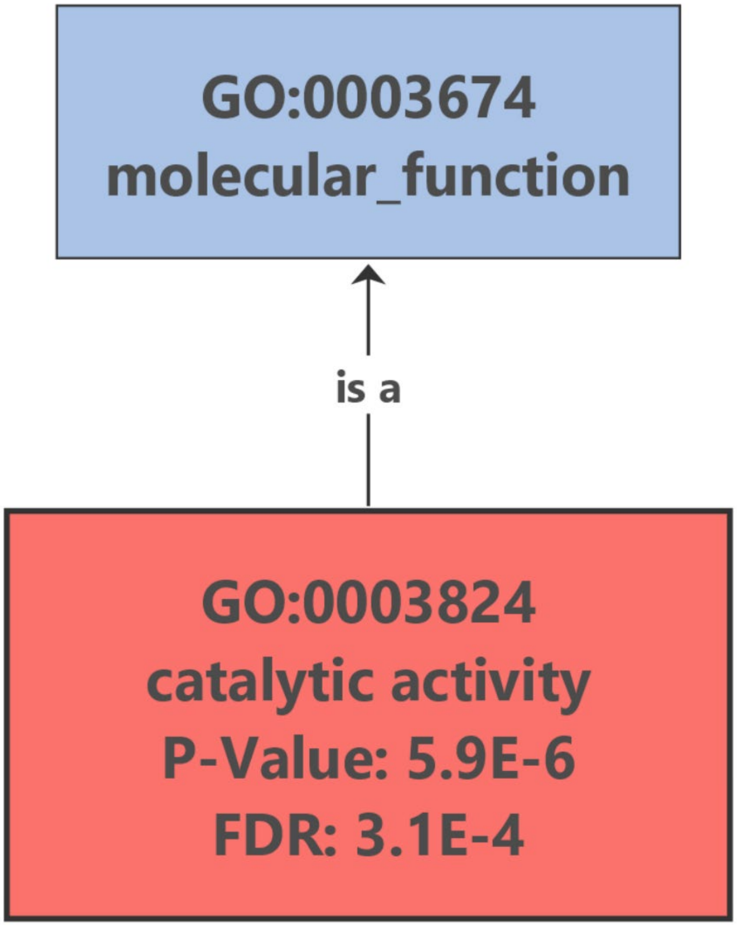
GO:0003674
molecular_function
is a
GO:0003824
catalytic activity
P-Value: 5.9E-6
FDR: 3.1E-4

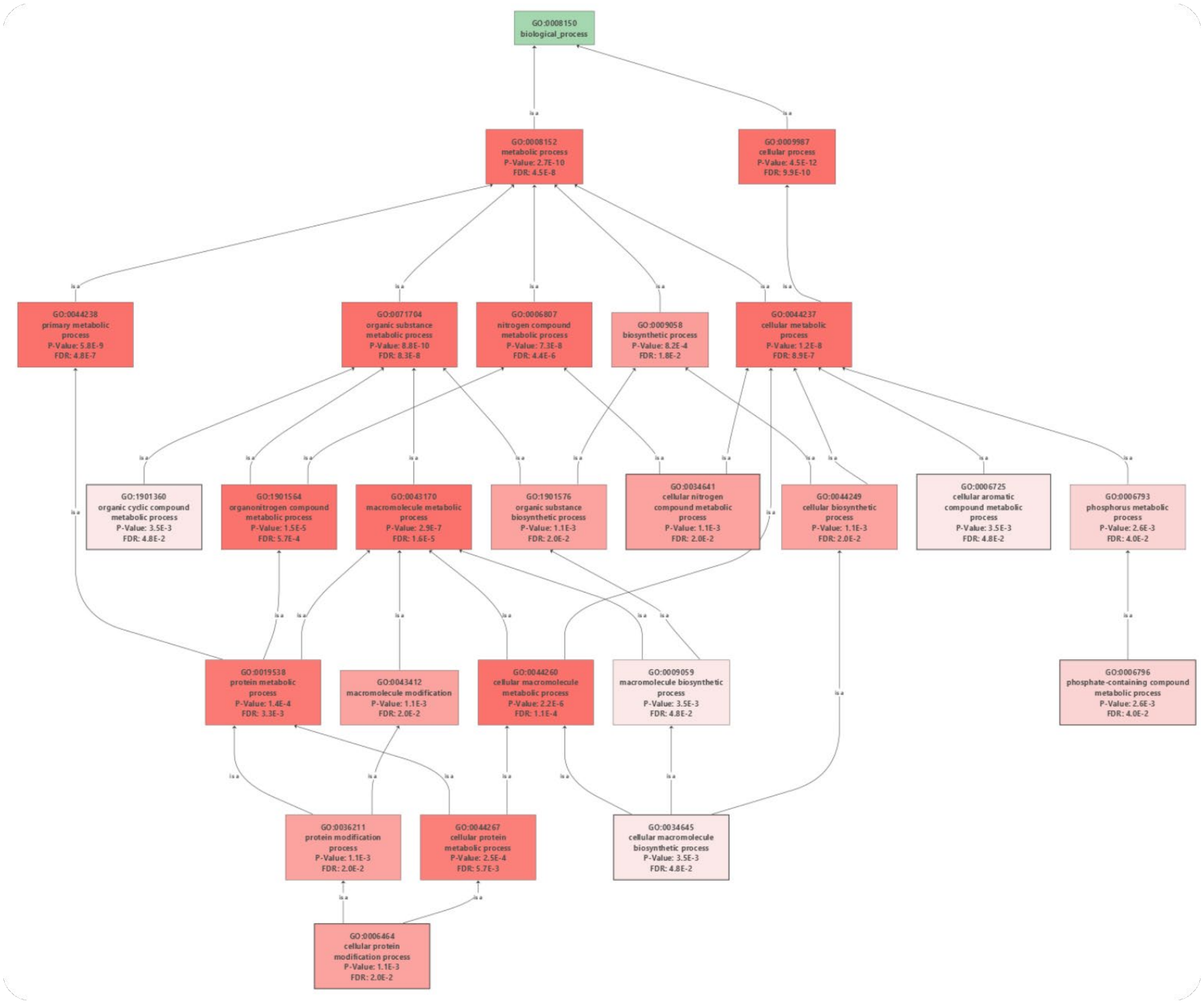
GO:0008150
biological_process
GO:0008152
metabolic process
P-Value: 2.7E-10
FDR: 4.5E-8
GO:0009987
cellular process
P-Value: 4.5E-12
FDR: 9.9E-10
GO:0044238
primary metabolic process
P-Value: 5.8E-9
FDR: 4.8E-7
GO:0071704
organic substance metabolic process
P-Value: 8.8E-10
FDR: 8.3E-8
GO:0006807
nitrogen compound metabolic process
P-Value: 7.3E-8
FDR: 4.4E-6
GO:0009058
biosynthetic process
P-Value: 8.2E-4
FDR: 1.8E-2
GO:0044237
cellular metabolic process
P-Value: 1.2E-8
FDR: 8.9E-7
GO:1901360
organic cyclic compound metabolic process
P-Value: 3.5E-3
FDR: 4.8E-2
GO:1901564
organonitrogen compound metabolic process
P-Value: 1.5E-5
FDR: 5.7E-4
GO:0043170
macromolecule metabolic process
P-Value: 2.9E-7
FDR: 1.6E-5
GO:1901576
organic substance biosynthetic process
P-Value: 1.1E-3
FDR: 2.0E-2
GO:0034641
cellular nitrogen compound metabolic process
P-Value: 1.1E-3
FDR: 2.0E-2
GO:0044249
cellular biosynthetic process
P-Value: 1.1E-3
FDR: 2.0E-2
GO:0006725
cellular aromatic compound metabolic process
P-Value: 3.5E-3
FDR: 4.8E-2
GO:0006793
phosphorus metabolic process
P-Value: 2.6E-3
FDR: 4.0E-2
GO:0019538
protein metabolic process
P-Value: 1.4E-4
FDR: 3.3E-3
GO:0043412
macromolecule modification
P-Value: 1.1E-3
FDR: 2.0E-2
GO:0044260
cellular macromolecule metabolic process
P-Value: 2.2E-6
FDR: 1.1E-4
GO:0009059
macromolecule biosynthetic process
P-Value: 3.5E-3
FDR: 4.8E-2
GO:0006796
phosphate-containing compound metabolic process
P-Value: 2.6E-3
FDR: 4.0E-2
GO:0036211
protein modification process
P-Value: 1.1E-3
FDR: 2.0E-2
GO:0044267
cellular protein metabolic process
P-Value: 2.5E-4
FDR: 5.7E-3
GO:0034645
cellular macromolecule biosynthetic process
P-Value: 3.5E-3
FDR: 4.8E-2
GO:0006464
cellular protein modification process
P-Value: 1.1E-3
FDR: 2.0E-2

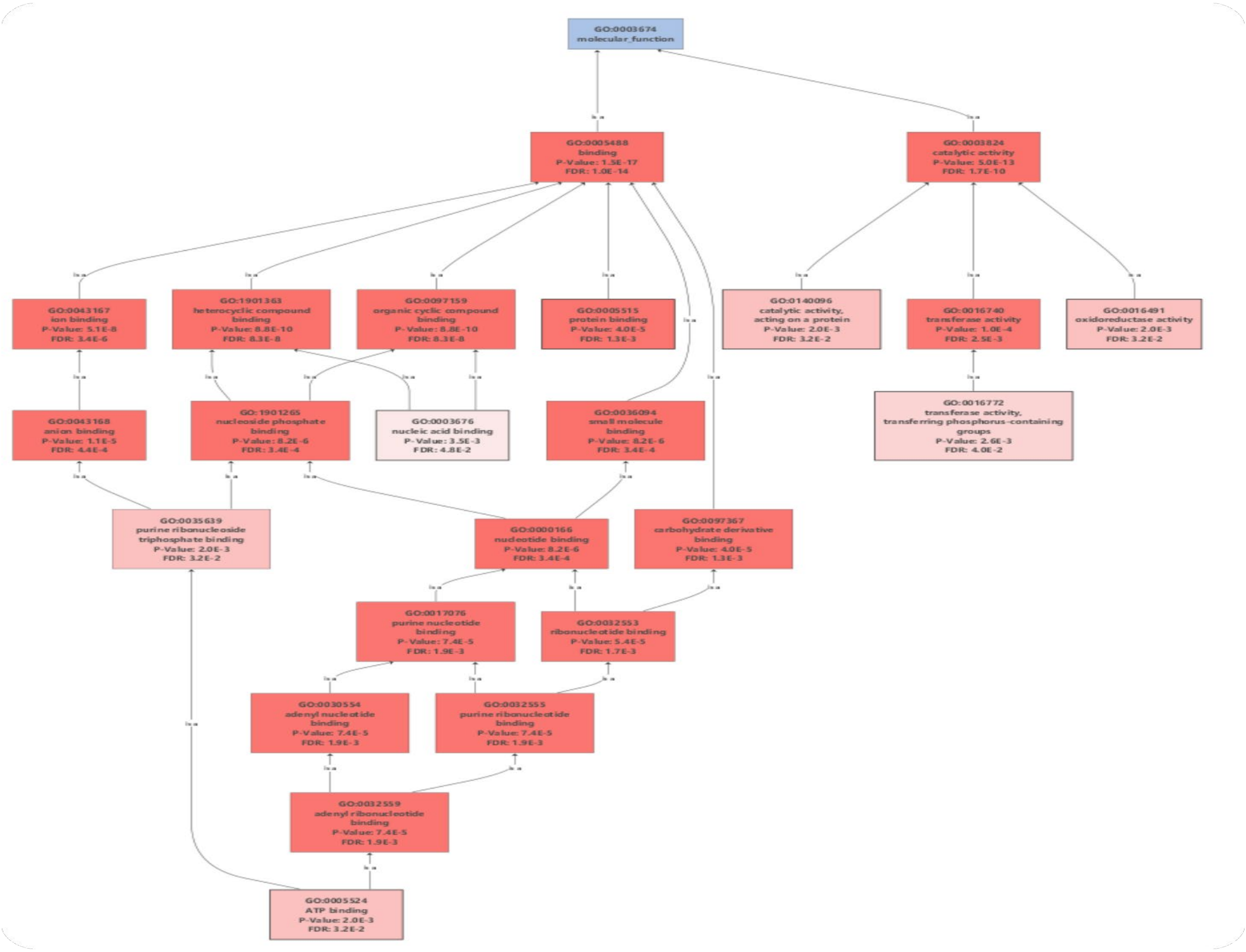

Supplementary Figure S11. GO enrichment analysis of molecular function (mf) in SESE. All GO terms are with p-value and FDR, calculated by Blast2GO.
